# Phenotypic Characterization and Pulsed-Field Gel Electrophoresis and Random Amplified Polymorphic DNA-PCR Profiling of *Erysipelothrix rhusiopathiae* Isolated from Erysipelas in Domestic Geese in Poland (2008–2018)

**DOI:** 10.3390/vetsci12121202

**Published:** 2025-12-15

**Authors:** Kamila Bobrek, Andrzej Gaweł

**Affiliations:** Department of Epizootiology and Clinic of Bird and Exotic Animals, Faculty of Veterinary Medicine, Wrocław University of Environmental and Life Sciences, pl. Grunwaldzki 45, 50-366 Wrocław, Poland; kamila.bobrek@upwr.edu.pl

**Keywords:** *Erysipelothrix rhusiopathiae*, geese, genetic analyses, biochemical features, genetic characteristics, PFGE, RAPD

## Abstract

*Erysipelothrix rhusiopathiae* is a small Gram-positive rod causing erysipelas in many animal species and humans. In poultry, disease which takes an acute form with high mortality is noted mostly in turkey, hen, and goo se flocks. Especially in geese, erysipelas cause high economic losses. In this study, we determined the phenotypic and genotypic features of *Erysipelothrix* strains isolated from diseased geese, using the API Coryne tests for biochemical characteristics and PCR, random amplified polymorphic DNA-PCR (RAPD) and pulsed-field gel electrophoresis (PFGE) for genetic characteristics. The isolates were confirmed with PCR to be *E. rhusiopathiae* and belonged to serotypes 1b, 2, and 5 with 1b serotype domination (55.3%). Among 47 isolates, five RAPD profiles (marked as A–E) and 7 PFGE profiles (marked I–VII) were noted. The RAPD profiles contained four to six bands and the PFGE profiles nine to eleven bands. The most common RAPD profile was B profile (42.5%), and the most common PFGE profile was I (36.2%). Five biochemical types of *E. rhusipathiae* were identified with the most common biochemical type (pyrrolidonyl arylamidase, acetyl-b-glucosaminidase, glucose, ribose, lactose- positive), which included 68.1% isolates and was matched to *E. rhusiopathiae* in 99.9%. Differences in biochemical reactions among the strains were related to the ability to degrade pyrazinamidase, alkaline phosphatase, and ribose. This study shows that most *E. rhusiopathiae* strains isolated from geese differ in their phenotypic and molecular characteristics, and there are no distinctive features that are typical of strains from geese.

## Simple Summary

*Erysipelothrix rhusiopathiae* is a bacterium causing erysipelas in many animal species and humans. Erysipelas in poultry usually takes the acute form, with high mortality in the flock causing economic losses for poultry producers, especially geese farmers. Because there are not much data on the *E*. *rhusiopathiae* strains featured in geese, we characterized 47 previously collected *Erysipelothrix* strains using the molecular (random amplified polymorphic DNA-PCR—RAPD and pulsed-field gel electrophoresis—PFGE) and biochemical methods. The bacteria isolates belonging to three serotypes (1b, 2, and 5) showed five biochemical types, five RAPD profiles (named A–E), and seven PFGE profiles (named I–VII). The most common RAPD profile was noted at 42.5% of isolates and the PFGE’s most common profile at 36.2%. The most common biochemical type included 68.1% isolates and was matched to *E. rhusiopathiae* in 99.9%. Differences in biochemical types among the strains concerned three reactions. Our research showed that isolates from the erysipelas in geese outbreaks had different biochemical and genetic characteristics. There was no profile which could be named as typical for erysipelas in goo se cases.

## 1. Introduction

*Erysipelothrix rhusiopathiae* is a small Gram-positive rod causing erysipelas in many animal species and humans. The disease is most commonly found in pigs, but erysipelas cases have also been reported in domesticated and wild mammals, birds, fish, and reptiles [1,2,3]. In the past, the disease outbreaks of economic significance in poultry were infrequently reported in species other than turkeys [4,5,6], but recently, outbreaks with high mortality in laying hens and geese have also been reported by a few European countries [7,8,9,10,11,12]. Erysipelas cases were also noted in other bird species, such as emus, kakapo, quails, pheasants, penguins, and pigeons [1,2,13,14,15,16,17,18,19].

The name of the bacterium comes from the Greek words erysipelas (red skin) and trix (the word for hair), and the species name is from rhusius—redness, and pathus—disease. The comparison to hair comes from a specific image of the bacterium on a glass slide—long, filamentous cells resembling hair, which showed rough colonies from a chronic form of the disease. The rough form (R) has been isolated on blood agar from tissues of animals with chronic disease. Rough colonies are approximately 1–2 mm in size, have irregular margins, and are flat and dry. The second form of colony is a smooth colony (S) which is small, 0.3–1.5 mm in diameter, circular, and slightly convex. *Erysipelothrix* smooth colonies tend to grow in the acute form of the disease, forming small colonies with a zone of alpha hemolysis [1,2]. Virulence is associated with the production of a protective polysaccharide capsule. Host antibodies directed against the protein encoded by the surface protective antigen (*Spa*) genes of *Erysipelothrix* spp. are known to protect against infection [2,3].

*E. rhusiopathiae* infections occur through damaged skin or mucous membranes and hematophagous parasite transmission. Oral and intramuscular infection has been experimentally confirmed. The number of bacteria in the blood at the time of the first symptoms of the experimental disease in turkeys ranged from 10^7^ to 10^8^/mL [5]. Although the infectious dose of the pathogen was not known in field cases, some authors reported an exacerbation of the disease in older birds [9,20]. It is confirmed that the housing system is a crucial factor in erysipelas outbreaks—flocks with outdoor access are most susceptible to the disease [3,7,10,21].

Erysipelas in poultry usually take the acute form with high mortality in the flock. Outbreaks usually occur suddenly, initially with a few dead birds found, followed by an increase in mortality over the following days. Mortality rates can range from <1% to 50% [1,6,14]. The lack of specific clinical symptoms preceding death is often noted [10]; however, birds may show depression and an unsteady gait, ruffling of feathers or reduced food intake [1,2,3,22]. Sometimes, as a result of fighting, male turkeys may develop swollen and purple snoods and cutaneous lesions. Turkey hens may present the perineal congestion and skin discoloration four to five days after artificial insemination [2,3].

Most infected birds die in the acute stage of erysipelas, so the body condition is good. The skeletal muscles are congested and petechiae are visible on the subcutaneous and visceral fat [3,6,10]. The gross lesions are noted in multiple organs such as the liver, heart, spleen, and lungs. The parenchymal organs are swollen and congested, and petechiae are observed on the epicardium, pancreas, and liver of birds. The liver and spleen are dark and friable. In laying birds, free yolk mass is often found in the coelomic cavity [4,5,10,11].

In birds that survive the acute stage, endocarditis, synovitis, and arthritis may develop. The chronic form, with joint inflammation, is rarely noted, mostly in vaccinated animals [4,5,6].

The course of avian erysipelas is well known in terms of clinical and post-mortem changes and treatment methods, but information on the characteristics of isolates is scarce. Currently available data on the genetic characteristics of *E. rhusiopathiae* isolates, other than swine origin, are missing [23,24,25].

In this study, we determined the phenotypic and genotypic features of *Erysipelothrix rhusiopathiae* isolates from farmed geese in Poland in 2008–2018. We assumed that there were some common features for *E. rhusiopathiae* isolates and a typical strain causing erysipelas in geese.

## 2. Materials and Methods

### 2.1. Isolation and Identification of Erysipelothrix Strains

#### 2.1.1. Geese Necropsy and Erysipelothrix Isolation

All necropsied geese came from commercial farms, which provided animals free access to paddocks. Necropsies of dead geese and future investigation which depended on the observed lesions were performed at the Department of Epizootiology with the Clinic of Birds and Exotic Animals, Wroclaw Environmental and Life Sciences, in cooperation with poultry veterinarians.

*Erysipelothrix* strains were isolated post-mortem from geese with lesions that were typical for septicemia, as described previously [25]. The predominant lesions were various sized petechiae on the epicardium, pancreas, and liver. The lungs’ edema and congestion was also observed. The organs with visible lesions (liver, heart, lungs) were taken for microbiological tests. Each sample was cultured on typical media for 24 h at 37 °C under aerobic conditions. Cultures growing on blood agar plates (Graso, Owidz, Poland) as small, grayish, translucent colonies with the morphology of slender Gram-positive rods were suspected *Erysipelothrix* and were tested with genera and species primers.

#### 2.1.2. Genetic Identification of the Erysipelothrix Strains

DNA from each isolate was extracted using the Genomic Mini Kit (A&A Biotechnology, Gdynia, Poland), according to the manufacturer’s instructions. To determine the species and serotype of the *Erysipelothrix* isolates, genomic DNA was used for PCR amplification with MO101-MO102 primers (Genomed, Warsaw, Poland), identifying the genus, ER1, ER2, ER3, and ER4 primers specific for the species, and primers typical for 1a, 1b, 2, and 5 serotypes of *Erysipelothrix* [Table 1 and Table 2].

PCRs were performed in a 25 µL reaction mixture containing 5 ng template DNA, 10 pmol of each primer, 12.5 µL PCR Mix Plus (1.25 U) (A&A Biotechnology, Gdynia, Poland) and ultrapure, nuclease free water in the conditions described previously [13]. For specificity control, as a positive control sample, *E. rhusiopathiae* IW445 from the National Veterinary Research Institute’s (Puławy, Poland) was isolated from erysipelas case in swine, and as a negative control sample, *E. coli* ATCC 25922 and *S. aureus* ATCC 29213 from our collection were used.

The PCR products were separated by electrophoresis in 1.5% agarose gel stained with CybrGreen (Sigma Aldrich, Poznan, Poland). The representative products of the reaction with genus-, species-, and serotype-specific primers were selected and sent to Macrogen (Amsterdam, The Netherlands) for Sanger sequencing with both forward and reverse PCR primers, as described previously [25]. The isolates confirmed as *E. rhusiopathiae* collected between 2008 and 2018 were preserved and stored at −80 °C in 2 mL tubes with 40% glycerol/10 mL for future tests.

### 2.2. Genetic Characteristics of Erysipelothrix Isolates

#### 2.2.1. Random Amplified Polymorphic DNA-PCR (RAPD)

The RAPD with NK6 (5′-CCCGCGCCCC-3′) and NK 51 (5′-GGTGGTGGTATC-3′) primers (Genomed, Warsaw, Poland) were performed [29] in a 25 µL reaction mixture with 10 ng template DNA, 10 pmol of each primer, 12.5 µL PCR Mix Plus (1.25 U) (A&A Biotechnology, Gdynia, Poland), and ultrapure water. The cycling program was 4 cycles at 94 °C for 5 min, 34 °C for 5 min, and 72 °C for 5 min; 30 cycles at 94 °C for 1 min, 34 °C for 1 min, and 72 °C for 2 min; with final elongation for 10 min at 72 °C with the use of iCycler (Biorad Laboratories Inc., Hercules, CA, USA). The PCR products were separated by electrophoresis in 1.5% agarose gel stained with CybrGreen (Sigma-Aldrich Co., Poznan, Poland).

#### 2.2.2. Pulsed-Field Gel Electrophoresis (PFGE)

PFGE was carried out according to the method of Opriessnig et al. [30]. The bacteria strains were cultured overnight on blood agar plates and next, picked with a swab and suspended in a cell-suspension buffer. The absorbance of the suspension was 1.0 in a spectrophotometer set at a wavelength of 612 nm. To the 200 µL bacterial suspension, 0.5 U of lysozyme (A&A Biotechnology, Gdansk, Poland) were added and incubated for 30 min at 37 °C. After incubation, 0.225 U mutanolysin (Sigma-Aldrich Co., Poznan, Poland), 0.2 mg proteinase K (A&A Biotechnology, Gdansk, Poland), and 200 µL melt plug agar (Agarose Reducta, Prona, Burgos, Spain) were added and left to solidify in agarose plug molds for 10 min in 5 °C.

The solidified plugs were digested in a cell lysis buffer with 40 µL proteinase K (A&A Biotechnology, Gdansk, Poland) in a shaking water bath at 54 °C for 1.5 h, and after washing, each plug was incubated with 0.5% Tango buffer at 30 °C for 1 h. The last step was digestion with SmaI at a working concentration of 2.5% and 10% buffer, at 30 °C for 2 h.

The digested plugs were loaded in their appropriate wells in the pulsed-field certified agarose (Agarose Technica, Prona, Burgos, Spain). Electrophoresis was conducted in 0.5× Tris/Borate/EDTA Buffer (TBE) (Sigma-Aldrich Co., Poznan, Poland) in a contour-clamped homogeneous electric field (CHEF DRIII, Biorad Laboratories Inc., Hercules, CA, USA) for 21 h at 12 °C and 6 V with pulse times from an initial 2.2 s to a final 64 s. PFGE patterns were detected by UV transillumination after ethidium bromide staining. CHEF DNA Size Standard, 48.5–1000 kb Lambda ladder (Biorad Laboratories Inc., Hercules, CA, USA) was used as the DNA size standard.

### 2.3. Biochemical Characteristics of the Isolates

Biochemical identification was performed using the commercial API Coryne test (bioMérieux, Lyon, France), according to the manufacturer’s instructions. The test contains tubes with reagents for nitrates (NIT), pyrazinamidase (PYZ), pyrrolidonyl arylamidase (PYR A), alkaline phosphatase (PAL), beta glucuronidase (βGUR), beta galactosidase (βGAL), alpha glucosidase (αGLU), N-Acetyl-beta glucosaminidase (βNAG), aesculin (ESC), urease (URE), and gelatin (GEL), which are enzymatic tests, and glucose (GLU), ribose (RIB), xylose (XYL), mannitol (MAN), maltose (MAL), lactose (LAC), sucrose (SAC), and glycogen (GLYG), which are fermentation tests. The catalase test (CAT) is made by adding 1 drop of hydrogen peroxide to the gelatin or aesculin tube. In addition to the test, the product also includes ampoules with medium and turbidity control. The inoculum for enzymatic tests was prepared in distilled water and for fermentation tests in the attached medium, according to the manufacturer’s instructions. From the results of individual reactions (positive or negative), a seven -digit numerical profile was obtained and read by the bioMerieux Center.

## 3. Results

### 3.1. Isolation and Identification of Erysipelothrix Strains

The morphology of bacterial cells was typical for *Erysipelothrix* sp., and all of them were Gram-positive rods.

All 47 strains collected between 2008 and 2018 from geese septicemia cases were identified by PCR with genus- and species-specific primers as *E. rhusiopathiae*, as described previously [25]. Among the strains tested, isolates 1–7 came from 2008, 8–21 from 2009, 22–24 from 2010, 25–26 from 2011, 27–33 from 2012, 34–37 from 2013, 38–42 from 2014, 43–44 from 2015, 45–46 from 2016, and 47 from 2018.

No *E. tonsillarum, Erysipelothrix* sp. strain 1, or *Erysipelothrix* sp. strain 2 were detected. The most prevalent serotype was serotype 1b (26 isolates; 55.3%), then serotype 2 (13 isolates; 27.7%), and the least common was serotype 5 (8 isolates; 17%) [Table 3].

### 3.2. Genetic Characteristics of Erysipelothrix Isolates

#### 3.2.1. Random Amplified Polymorphic DNA-PCR (RAPD)

Using the NK6 primer, eight products were obtained. The product sizes were 1950, 1650, 1300, 1150, 1100, 1050, 700, and 350 bp. This primer produced five RAPD patterns designated as RAPD profiles A through to E. The most commonly noted was the B profile, which included 20 isolates, which gives 42.5%. This profile identified four products with approximate sizes of 700–1950 bp. Profile E showed one additional product of approximately 350 bp compared to profile B (five bands: 350–1950 bp) and was represented by five *E. rhusiopathiae* isolates (10.6%). The profile D, which included six isolates (12.8%), showed the presence of six bands (350–1950 bp) and differed from profile E, just one product of approximately 1200 bp. Genetic profile A, with five bands (700–1950 bp), was the second most common profile (27.7%, 13 strains) and differed from profile B by the presence of a 1050 bp product. Genetic profile C, represented by three isolates (6.4%), had six products of 700–1950 base pairs (Table 4, Supplementary Figures S1 and S2).

For NK51 primer, just one product (~800 bp) was obtained for all examined *E. rhusiopathiae* strains, although other authors have also shown different patterns using this primer.

#### 3.2.2. Pulsed-Field Gel Electrophoresis (PFGE)

PFGE patterns after restriction with SmaI were characterized by 9–11 bands in a 38–437 kb size range. With PFGE, it was possible to distinguish seven different patterns [Table 5, Supplementary Figures S1 and S2].

Seventeen isolates showed pattern I with nine bands, with approximate sizes of 58–402 kb, which gives 36.2% of all strains. The next most frequently reported profiles, both with 10 bands, were patterns III (58–402 kb) with 17% of isolates and VII (42–309 kb) with 14.9% of isolates. The largest number of patterns showed profile II with 11 bands (38–263 kb), which included 8.5% isolates (four strains). Three profiles which consisted of 10 bands were profiles IV, V, and VI, and contained 6.4%, 10.6%, and 6.4% isolates, respectively. The band sizes of profile IV were between 58 and 402 kb, V-58 and 437 kb and VI-44 and 402 kb.

### 3.3. Biochemical Characteristics of the Isolates

Of the 47 tested strains, 41 were identified as *E. rhusiopathiae*, with over 90% identification accuracy. Biochemical type, described by code 4020340, confirming a 99.9% match to *E. rhusiopathiae*, was obtained by testing 32 strains (68.1%). Two strains (4.3%) were identified with 98.1% species identity, receiving code 4120140, and seven strains (14.9%) were identified with 92.6% identity, receiving code 4120340. Five strains (10.6%) of *E. rhusiopathiae* were identified based on biochemical characteristics as *Arcanobacterium haemoliticum*, but *E. rhusiopathiae* was listed as a second possible taxon [Table 3]. Code 4520340 identified *E. rhusiopathiae* at a 17.4% rate and applied to one strain (2.1%), while code 6120340 identified *E. rhusiopathiae* at a 10.6% rate and applied to five strains tested (10.5%). Strains classified as *E. rhusiopathiae* with 99.9% agreement demonstrated the ability to degrade pyrrolidonyl arylamidase (PYR A), acetyl-b-glucosaminidase (βNAG), glucose (GLU), ribose (RIB), and lactose (LAC); the remaining test results were negative. Differences in biochemical reactions were observed in the ability to degrade pyrazinamidase (PYZ), alkaline phosphatase (PAL), and ribose (RIB). Compared to strains identified with code 4020340, strains with code 4120340 demonstrated the ability to degrade PAL, and strain 6120340 demonstrated the ability to degrade PYZ and PAL. Strains with identification code 4120140 showed the ability to degrade PAL and no RIB fermentation. Strain code 4520340 degraded PAL and betaGAL [Table 6].

## 4. Discussion

Erysipelas is a significant health problem occurring in farmed geese, and information on the characteristics of the *E. rhusiopathiae* strains causing this disease is limited; thus, our research provides new information on this topic [12,31]. Our study represents the first biochemical and genetic (RAPD and PFGE) analysis of geese isolates of *E. rhusiopathiae.* Diagnosis of erysipelas is typically confirmed by culture of the causative agent and is sometimes supported by PCR reaction. It was confirmed that erysipelas in geese is caused only by *E. rhusiopathae*; there was no *E. tonsillarum* isolation. Our research demonstrated that the phenotypical characterization in most cases of *E. rhusiopathiae* is useful, but in some cases, could be mistaken. The biochemical tests on the geese strains show that it could be a method for identifying *E. rhusiopathiae*, but some of the isolates could be wrongly classified. The reactions that are a typical for *E. rhusiopathiae* are positive tests for pyrazinamidase and alkaline phosphatase or alkaline phosphatase and beta galactosidase. Dunbar and Cla rridge [32] also observed that some of the *E. rhusiopathiae* strains isolated from human patients could give a typical API results. One of the four (25%) isolates was alkaline phosphatase positive, when in geese strains, 14.9% of isolates showed that biochemical type. The pyrrolidonyl arylamidase negative profile noted in the patient was not detected in geese.

RAPD and PFGE methods have proven to be reliable tools for differentiating species and strains of a single genus, as well as for use in epidemiological studies of many pathogenic bacteria [33,34,35]. PFGE is considered to be the gold standard, because the profiles can be compared and exchanged between laboratories and the method is an inexpensive and relevant technique in bacteria typing compared to whole genome sequencing [35,36,37].

In our study, PFGE showed higher viability to distinguish different isolates of *E. rhusiopatihiae* than RAPD, but both methods are useful. No correlation between RAPD profiles and serotypes was noted. Similarly, Hassainen et al. [38] demonstrated that the strains they studied, classified as serotypes 1 and 2, showed the same banding pattern. Studies by Okatani et al. [17] demonstrated that strains classified as a single serotype can produce different electrophoretic profiles. This indicates a lack of correlation between banding pattern and serotype. The correlation between RAPD profiles and *Erysipelothrix* species was confirmed by Okatani et al. [29], showing that in the RAPD-PCR reaction, using the NK 51 primer shows four electrophoretic profiles specific for the species *E. rhusiopathiae* (one band ~0.88 kbp)*, E. tonsillarum (*~1.2 kbp), and *E. rhusiopathiae* serovar 13 (~0.65 kbp) and serovar 18 (clear band of ~0.42 kbp and three weak bands of ~1.26 kbp, 0.92 kbp, and 0.44 kbp). Our research showed just one profile for all isolates with a single band ~0.8 kbp, which corresponds with the *E. rhusiopathiae* profile.

In the RAPD with NK6 primer, we described five profiles, among which profile B (with four bands) had a similar pattern to isolate R32E11, belonging to serotype 2, whose origin was not documented [29]. The remaining genetic profiles, showing five or six band patterns, did not resemble the previously described types [29,38], but it is believed that the RAPD-PCR method is not very reproducible—the results obtained are influenced by many factors and may vary depending on the laboratories where the analysis is performed. In contrast to the RAPD method, PFGE is more repeatable, regardless of the laboratory [37]. Unfortunately, just a few articles contain the view of *Erysipelothrix* PFGE profiles [8,29,30,38,39], and the comparison of PFGE patterns is difficult.

In our study, seven PFGE profiles based on the restricted DNA bands were noted. Most of the isolates showed profile I, which was unique compared to other researchers’ results. The genetic profile designated as III in our study was similar in terms of the number and size of restriction fragments to that of *E. rhusiopathiae* from pigs designated as 1B in the work of Opriessnig et al. [30]. The profile marked as VIII, on the other hand, was similar in terms of the size of restriction fragments to the genetic profile of the pig strain 422/1E1 described by Okatani et al. [35].

The *Erysipelothix* PFGE patterns demonstrated in the manuscript’s pictures are varied. The fewest band profiles of *Erysipelothrix* strains were obtained in isolates from white-lipped peccary (Tayassu pecari), which contained eight or nine bands [40]. The lambs’ isolates showed 9–11 band profiles [39], which was consistent with our results, but any of the patterns were similar to geese profiles. The swine isolates obtained from US swines showed PFGE profiles characterized by 8–14 bands. The greatest diversity of the profiles (resulting from the number of tested *Erysipelothrix* isolated from different animal species) was shown by Okatani et al., where the number of bands in the PFGE profiles ranged from 8 to 27. The bird s’ origin isolates, including chicken and parrot, showed 10–14 band PGFE patterns. The profiles with the highest number of bands (exceeding 20) came from the wild boar and deer meat and porcine spleen.

The present study shows that most of the *E. rhusiopathiae* strains isolated from geese are different in phenotypic and molecular characterization, and there are no distinctive features typical of strains from geese. Our previous biochemical and genetic analysis of the isolates collected during two erysipelas outbreaks in geese from one farm indicated that the strains varied, and they might be from different sources of the infection [31].

## 5. Conclusions

Erysipelas in geese is caused by *E. rhusiopathiae* strains that differ in biochemical and genetic characteristics. Further research into the molecular characteristics of isolates, immunological aspects, and effective erysipelas prevention in birds is needed.

## Figures and Tables

**Table 1:** Primers for PCR reactions, identifying genus and species of *Erysipelothrix*.

	Primers Name	Sequence	Targeting	Product Size [bp]	References
Primers for genus and species identification	MO101-MO102	5′-AGATGCCTAGAAACTGTA-3′ 5′-CTGTATCCGCCATAACTA-3′	*Erysipelothrix* spp.	407	[26]
	ER1F-ER1R	5′-GTTCATCTCTCTAATGCACTAC-3′ 5′-TGTTGGACTACTAATCGTTTCG-3′	*E. rhusiopathiae*	339	[27]
	ER2F-ER2R	5′-ATGTAATATGATCTGGTGATTTG-3′ 5′-AGGACTGCTGATTGTCTCATG-3′	*E. tonsillarum*	384	[27]
	ER3F-ER3R	5′-TGGAGGACCGAACCGACTG-3′ 5′-AATTTTGGGACCTTAACTGGC-3′	*Erysipelothrix* sp. strain 1	289	[27]
	ER4F-ER4R	5′-TAAAGCACTAAGATCTGGTGG-3′ 5′-TCGGACTACTAATTGTCTCAG-3′	*Erysipelothrix* sp. strain 2	387	[27]

**Table 2:** Primers for PCR reactions identifying serotypes of *Erysipelothrix*.

	Primers Name	Sequence	Targeting	Product Size [bp]	References
Primers for serotype identification	1aF-1aR	5′-CTCCTAACGCTTTAGCACGC-3′ 5′-TGATCCTTTGCCACTAATGC-3′	*E. rhusiopathiae* serotype 1a	356	[28]
	1bF-1bR	5′-CGAAAGCATCCCTTAATCATTGC-3′ 5′-TGCGTGTAAAACCTGATCGTGTAAATC-3′	*E. rhusiopathiae* serotype 1b	1357	[28]
	2F-2R	5′-CCACGTCTTCCCACACTACAAAAAAGTAAATTC-3′ 5′-TCATCCTAATGCATATCATTATGTGGATATGAA-3	*E. rhusiopathiae* serotype 2	541	[28]
	5F-5R	5′-GCACGTTTCCAAATATTGTATCGAGTCT-3′ 5′-GAAATAATGCCGATAGATGGAGCACC-3′	*E. rhusiopathiae* serotype 5	194	[28]

**Table 3:** Characteristics of *E. rhusiopathiae* isolates.

Erysipelothrix Strain	Serotype	NK6 Profile	PFGE Profile	Biochemical Profile (API Code)
1	1b	A	II	6120340 *
2	2	B	II	6120340 *
3	1b	B	II	4020340
4	2	E	VII	4020340
5	2	B	I	4120340
6	5	B	I	4020340
7	1b	B	IV	6120340 *
8	1b	B	VI	6120340 *
9	1b	B	IV	4120340
10	5	A	I	4120340
11	1b	A	I	4020340
12	2	D	VII	4520340 *
13	1b	E	I	4020340
14	1b	E	I	4020340
15	1b	E	I	4020340
16	2	D	VII	4120140
17	5	A	V	4020340
18	2	B	I	4020340
19	1b	A	III	4020340
20	2	B	I	4020340
21	2	B	III	4020340
22	5	B	I	4020340
23	1b	A	III	4020340
24	1b	B	VII	4020340
25	1b	B	VII	4120340
26	2	C	I	4020340
27	1b	B	I	4020340
28	5	B	III	4020340
29	1b	A	V	4020340
30	1b	A	III	4020340
31	5	B	V	4120140
32	1b	A	III	4020340
33	1b	B	I	4020340
34	1b	A	I	4020340
35	1b	D	VI	4020340
36	1b	A	III	6120340 *
37	1b	E	II	4020340
38	2	D	I	4020340
39	2	C	VII	4020340
40	5	B	V	4020340
41	1b	A	I	4020340
42	1b	D	IV	4020340
43	2	C	III	4020340
44	5	A	V	4020340
45	1b	B	VI	4020340
46	2	B	I	4120340
47	1b	D	VII	4020340

* *Arcanobacterium haemoliticum* as a first possible taxon.

**Table 4:** The *E. rhusiopathiae* random amplified polymorphic DNA-PCR (RAPD) patterns with NK6 primer.

NK6 Profiles	Approximate RAPD Band Size [bp]	Number of Isolates [%]
A	700, 1050, 1300, 1650, 1950	13 [27.7]
B	700, 1300, 1650, 1950	20 [42.5]
C	700, 1050, 1100, 1300, 1650, 1950	3 [6.4]
D	350, 700, 1200, 1300, 1650, 1950	6 [12.8]
E	350, 700, 1300, 1650, 1950	5 [10.6]

**Table 5:** The *E. rhusiopathiae* PFGE patterns with SmaI restriction.

PFGE Profiles	Approximate Band Size [kb]	Number of Isolates [%]
I	58, 67, 96, 109, 127, 154, 205, 263, 402	17 [36.2]
II	38, 42, 67, 96, 109, 127, 154, 171, 205, 213, 263	4 [8.5]
III	58, 67, 83, 96, 109, 127, 154, 205, 227, 402	8 [17.0]
IV	58, 67, 83, 96, 109, 154, 171, 205, 263, 402	3 [6.4]
V	58, 67, 83, 96, 109, 127, 154, 205, 263, 437	5 [10.6]
VI	44, 58, 67, 83, 96, 140, 154, 171, 263, 402	3 [6.4]
VII	42, 58, 67, 83, 96, 140, 171, 205, 249, 309	7 [14.9]

**Table 6:** The biochemical characterization of the *E. rhusiopathiae* isolates.

		CODE			
REACTION	4020340	4120340	6120340	4120340	4520340
NIT	neg	neg	neg	neg	neg
PYZ	neg	neg	POS	neg	neg
PYR A	POS	POS	POS	POS	POS
PAL	neg	POS	POS	POS	POS
βGUR	neg	neg	neg	neg	neg
βGAL	neg	neg	neg	neg	POS
αGLU	neg	neg	neg	neg	neg
βNAG	POS	POS	POS	POS	POS
ESC	neg	neg	neg	neg	neg
URE	neg	neg	neg	neg	neg
GEL	neg	neg	neg	neg	neg
GLU	POS	POS	POS	POS	POS
RIB	POS	POS	POS	neg	POS
XYL	neg	neg	neg	neg	neg
MAN	neg	neg	neg	neg	neg
MAL	neg	neg	neg	neg	neg
LAC	POS	POS	POS	POS	POS
SAC	neg	neg	neg	neg	neg
GLYG	neg	neg	neg	neg	neg
CAT	neg	neg	neg	neg	neg

POS—positive reaction and neg—negative reaction.

## Data Availability

The original contributions presented in this study are included in the article/Supplementary Materials. Further inquiries can be directed to the corresponding author.

## Supplementary Materials

The following supporting information can be downloaded at: https://www.mdpi.com/article/10.3390/vetsci12121202/s1. Figure S1: RAPD profiles; Figure S2: PFGE profiles.

## References

[1] Bobrek K., Gaweł A., Mazurkiewicz M. (2013). Infections with *Erysipelothrix rhusiopathiae* in poultry flocks. World’s Poult. Sci. J..

[2] Eriksson H., Swayne D.E., Boulianne M., Logue C.M., McDougald L.R., Nair V., Suarez D.L. (2019). Erysipelas. Diseases of Poultry.

[3] Eriksson H., Wattrang E., Söderlund R., Jansson D.S. (2025). Erysipelas—A Review of an Emerging Disease in Layers. Avian Dis..

[4] Krasnodębska-Depta A., Koncicki A. (1988). Oral immunisation of turkey against red fever by means of the Orvac vaccine. Med. Weter..

[5] Bricker J., Saif Y. (1988). Use of live oral vaccine to immunize turkeys against erysipelas. Avian Dis..

[6] Brickford A., Corstvet R., Rosenwald A. (1978). Pathology of experimental erysipelas in turkeys. Avian Dis..

[7] Eriksson H., Nyman A.K., Fellström C., Wallgren P. (2013). Erysipelas in laying hens is associated with housing system. Vet. Rec..

[8] Eriksson H., Brannstrom S., Skarin H., Chirico J. (2010). Characterization of *Erysipelothrix rhusiopathiae* isolates from laying hens and poultry red mites (*Dermanyssus gallinae*) from an outbreak of erysipelas. Avian Pathol..

[9] Mazaheri A., Lierz M., Hafez H.M. (2005). Investigations on the pathogenicity of *Erysipelothrix rhusiopathiae* in laying hens. Avian Dis..

[10] Bobrek K., Nowak M., Borkowska J., Bobusia K., Gaweł A. (2016). An outbreak of erysipelas in commercial geese. Pak. Vet. J..

[11] Nowak T., Wódz K., Kwieciński P., Kwieciński A., Dec M. (2024). Incidence of erysipelas in waterfowl in Poland—Clinical and pathological investigations. Br. Poult. Sci..

[12] Dec M., Nowak T., Webster J., Wódz K. (2024). Serotypes, Antimicrobial Susceptibility, and Potential Mechanisms of Resistance Gene Transfer in *Erysipelothrix rhusiopathiae* Strains from Waterfowl in Poland. Int. J. Mol. Sci..

[13] Xie S., Hsu C.D., Tan B.Z.Y., Tay Y.H., Wong W.K. (2019). Erysipelothrix Septicaemia and Hepatitis in a Colony of Humboldt Penguins *(Spheniscus humboldti*). J. Comp. Pathol..

[14] Boerner L., Nevis K., Hinckley L., Weber E., Frasca S. (2004). *Erysipelothrix septicaemia* in a little blue penguin (*Eudyptula minor*). J. Vet. Diagn. Investig..

[15] Mutalib A., Keirs R., Austin F. (1995). Erysipelas in quail and suspected erysipeloid in plant employees. Avian Dis..

[16] Gartell B., Alley M., Mack H., Donald J., Mcinnes K., Jansen P. (2005). Erysipelas in the critically endangered kakapo (*Strigops habroptilus*). Avian Pathol..

[17] Milne E., Windsor R., Rogerson F., Pennycot T. (1997). Concurrent infection with enteric protozoa and *Erysipelothrix rhusiopathiae* in chicken and pheasant flocks. Vet. Rec..

[18] Morgan M., Britt J., Cockrill J., Eiten M. (1994). *Erysipelothrix rhusiopathiae* infection in an emu (*Dromaius novaehollandiae*). J. Vet. Diagn. Investig..

[19] Silva A.P., Cooper G., Blakey J., Jerry C., Shivaprasad H.L., Stoute S. (2020). Retrospective Summary of *Erysipelothrix rhusiopathiae* Diagnosed in Avian Species in California (2000–19). Avian Dis..

[20] Kurian A., Neumann E., Hall W., Marks D. (2012). Serological survey of exposure to *Erysipelothrix rhusiopathiae* in poultry in New Zealand. N. Z. Vet. J..

[21] Fossum O., Jansson D.S., Etterlin P.E., Vagsholm I. (2009). Causes of mortality in laying hens in different housing systems in 2001 to 2004. Acta Vet. Scand..

[22] Takahashi T., Takagi M., Yamaoka R., Ohishi K., Norimatsu M., Tamura Y., Nakamura M. (1994). Comparison of the patogenicity for chickens of *Erysipelothrix rhusiopathiae* and *Erysipelothrix tonsillarum*. Avian Pathol..

[23] Forde T.L., Kollanandi Ratheesh N., Harvey W.T., Thomson J.R., Williamson S., Biek R., Opriessnig T. (2020). Genomic and immunogenic protein diversity of *Erysipelothrix rhusiopathiae* isolated from pigs in Great Britain: Implications for vaccine protection. Front. Microbiol..

[24] Dec M., Łagowski D., Nowak T., Pietras-Ożga D., Herman K. (2023). Serotypes, Antibiotic Susceptibility, Genotypic Virulence Profiles and SpaA Variants of *Erysipelothrix rhusiopathiae* Strains Isolated from Pigs in Poland. Pathogens.

[25] Bobrek K., Gaweł A. (2023). Antimicrobial Resistance of *Erysipelothrix rhusiopathiae* Strains Isolated from Geese to Antimicrobials Widely Used in Veterinary Medicine. Antibiotics.

[26] Makino S., Okada Y., Maruyama T., Ishikawa K., Takahashi T., Nakamura M., Ezaki T., Morita H. (1994). Direct and rapid detection of *Erysipelothrix rhusiopathiae* DNA in animals by PCR. J. Clin. Microbiol..

[27] Takeshi K., Makino S., Ikeda T., Takada N., Nakashiro A., Nakanishi K., Oguma K., Katoh Y., Sunagawa H., Ohyama T. (1999). Direct and rapid detection by PCR of *Erysipelothrix* sp. DNAs prepared from bacterial strains and animal tissues. J. Clin. Microbiol..

[28] Shiraiwa K., Ogawa Y., Nishikawa S., Eguchi M., Shimoji Y. (2018). Identification of serovar 1a, 1b, 2, and 5 strains of *Erysipelothrix rhusiopathiae* by a conventional gel-based PCR. Vet. Microbiol..

[29] Okatani A.T., Hayashidani H., Takahashi T., Taniguchi T., Ogawa M., Kaneko K. (2000). Randomly amplified polymorphic DNA analysis of *Erysipelothrix* spp. *J*. Clin. Microbiol..

[30] Opriessnig T., Hoffman L.J., Harris D.L., Gaul S.B., Halbur P.G. (2004). *Erysipelothrix rhusiopathiae*: Genetic characterization of midwest US isolates and live commercial vaccines using pulsed-field gel electrophoresis. J. Vet. Diagn. Investig..

[31] Bobrek K., Gaweł A. (2015). Erysipelas Outbreaks in Flocks of Geese in Poland--Biochemical and Genetic Analyses of the Isolates. Avian Dis..

[32] Dunbar S.A., Clarridge J.E. (2000). Potential errors in recognition of *Erysipelothrix rhusiopathiae*. J. Clin. Microbiol..

[33] Gori A., Espinasse F., Deplano A., Nonhoff C., Nicolas M.H., Struelens M.J. (1996). Comparison of pulsed-field gel electrophoresis and randomly amplified DNA polymorphism analysis for typing extended-spectrum-β-lactamase-producing Klebsiella pneumoniae. J. Clin. Microbiol..

[34] Tynkkynen S., Satokari R., Saarela M., Mattila-Sandholm T., Saxelin M. (1999). Comparison of ribotyping, randomly amplified polymorphic DNA analysis, and pulsed-field gel electrophoresis in typing of *Lactobacillus rhamnosus* and *L. casei* strains. Appl. Environ. Microbiol..

[35] Okatani A.T., Uto T., Taniguchi T., Horisaka T., Horikita T., Kaneko K., Hayashidani H. (2001). Pulsed-field gel electrophoresis in differentiation of erysipelothrix species strains. J. Clin. Microbiol..

[36] Neoh H.M., Tan X.E., Sapri H.F., Tan T.L. (2019). Pulsed-field gel electrophoresis (PFGE): A review of the “gold standard” for bacteria typing and current alternatives. Infect. Genet. Evol..

[37] Félix B., Dao T.T., Grout J., Lombard B., Asséré A., Brisabois A., Roussel S. (2012). Pulsed-field gel electrophoresis, conventional, and molecular serotyping of Listeria monocytogenes from food proficiency testing trials toward an harmonization of subtyping at European level. Foodborne Pathog. Dis..

[38] Hassanein R., Suzuki Y., Sawada T. (2007). Epidemiological Analysis of Erysipelothrix Isolates from Various Animals by Acriflavine Resistance and RAPD Typing. J. Vet. Epidemiol..

[39] Ersdal C., Jørgensen H.J., Lie K.-I. (2014). Acute and Chronic *Erysipelothrix rhusiopathiae* Infection in Lambs. Vet. Pathol..

[40] Coutinho T.A., Moreno A.M., Imada Y., Lopez R., Neto J.S.F. (2012). Characterization of *Erysipelothrix rhusiopathiae* isolated from Brazilian *Tayassu pecari*. Trop. Anim. Health Prod..

