# Supplementary material for: Phenotypic Characterization and Pulsed-Field Gel Electrophoresis and Random Amplified Polymorphic DNA-PCR Profiling of Erysipelothrix rhusiopathiae Isolated from Erysipelas in Domestic Geese in Poland (2008–2018)
Source: Vet Sci. 2025 Dec 15;12(12):1202. doi: 10.3390/vetsci12121202 (PMC12737714; doi:10.3390/vetsci12121202)
Supplement: Supplementary file 1 [file vetsci-12-01202-s001.zip › vetsci-3877445-supplementary.pdf]

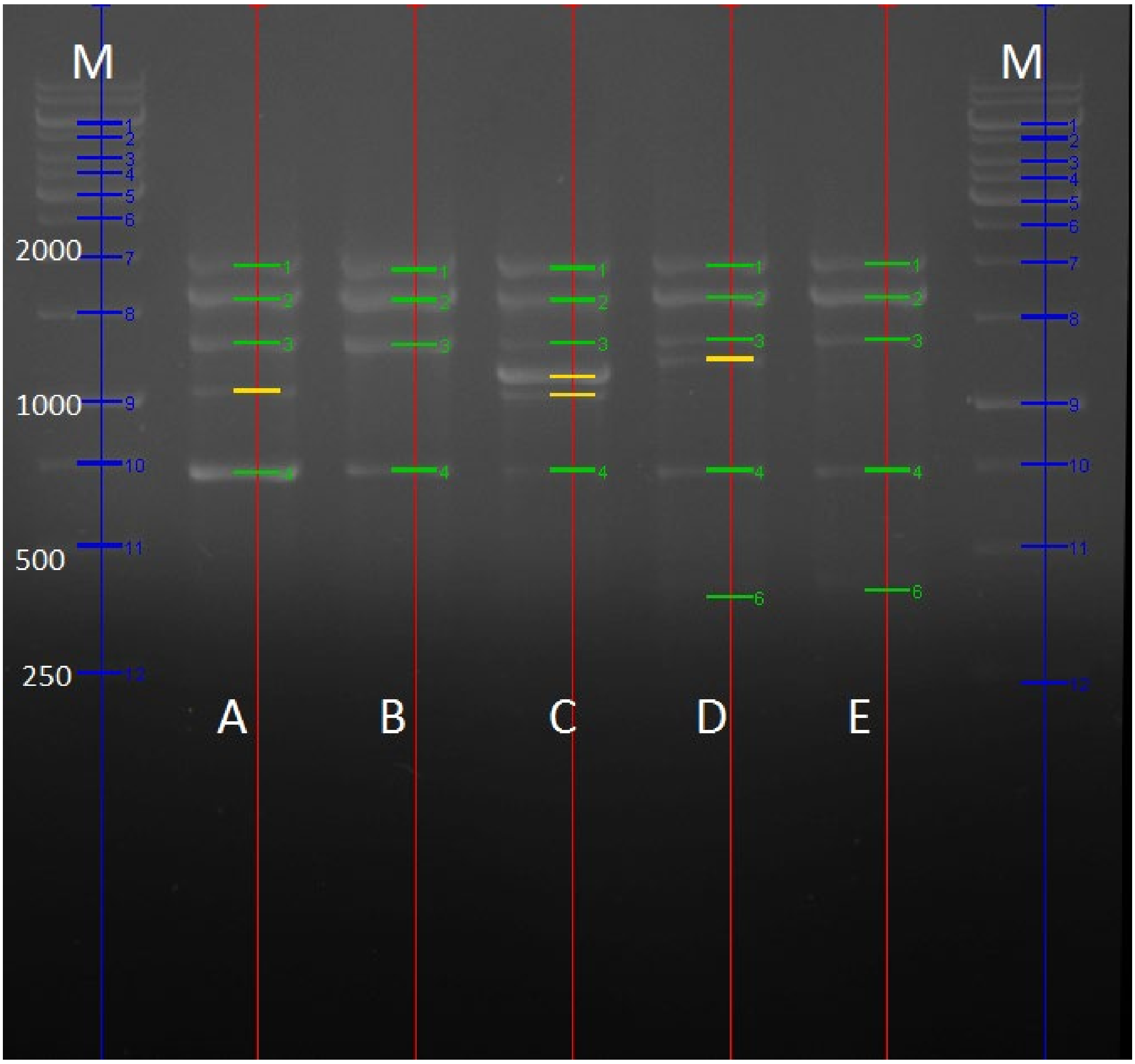

Figure S1: RAPD patterns obtained from 47 E.rhusiopathiae strains with the primer NK6. The lanes A to E are E. rhusiopathiae, lanes M, 1-kb ladder (GeneRuler 1kb DNA Ladder, ready-to-use; Thermo Fisher Scientific)

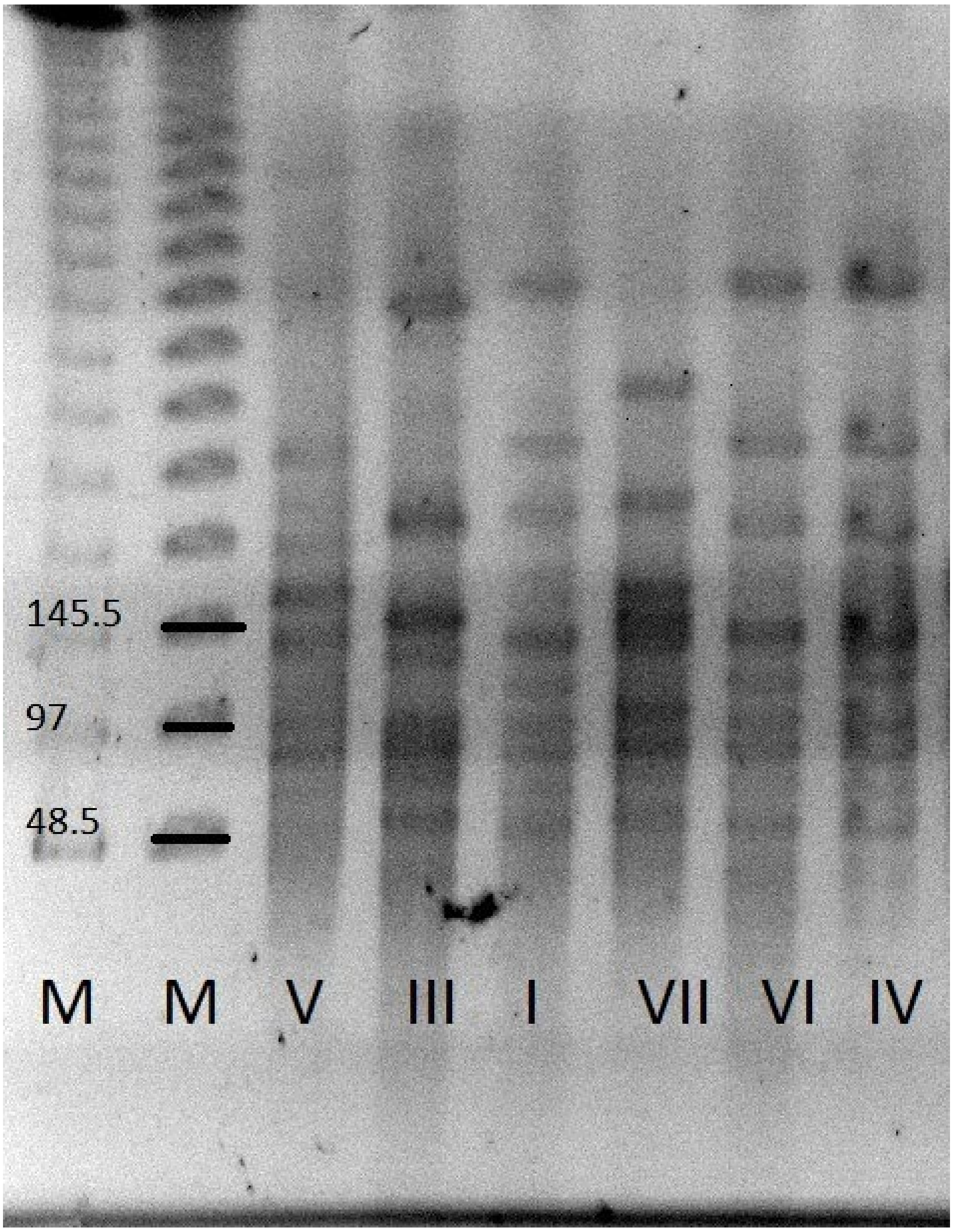

Figure S2: PFGE patterns produced from 47 E.rhusiopathiae strains by SmaI. Lanes I, III-VII are E. rhusiopathiae; lanes M, CHEF DNA Size Standard, 48.5–1,000 kb, Lambda Ladder (Bio-Rad)
